# Considerations on the Obstacles That Lead to Slow Recruitment in a Pain Management Clinical Trial: Experiences from the Belgian PELICAN (PrEgabalin Lidocaine Capsaicin Neuropathic Pain) Pragmatic Study

**DOI:** 10.1155/2023/7708982

**Published:** 2023-04-14

**Authors:** Guy H. Hans, Dima Almeshal, Lotte Vanlommel, Ella Roelant, Iris Verhaegen, Elke Smits, Koen Van Boxem, Robert Fontaine, The PELICAN Investigators Team

**Affiliations:** ^1^Multidisciplinary Pain Center, Antwerp University Hospital (UZA), Edegem, Belgium; ^2^ASTARC, University of Antwerp (UA), Antwerp, Belgium; ^3^Clinical Trial Center (CTC), Antwerp University Hospital (UZA), Edegem, Belgium; ^4^StatUa, Center for Statistics, University of Antwerp (UA), Antwerp, Belgium; ^5^Department of Anesthesiology, Intensive Care Medicine, Emergency Medicine and Pain Therapy, Hospital Oost-Limburg, Genk, Belgium; ^6^Multidisciplinary Pain Center, Vivalia, Libramont, Belgium

## Abstract

**Background:**

A qualitative evaluation study of the prematurely terminated PrEgabalin Lidocaine Capsaicin Neuropathic Pain (PELICAN) study was performed. The PELICAN study aimed to examine pain management for localized neuropathic pain (LNP), as epidemiological figures have shown a high percentage of LNP patients in Belgium. The study compared systemic and topical medications according to pain relief, adverse effects, and several measures of quality of life.

**Objective:**

Achieving better study patient recruitment through qualitative research. To investigate and determine the causes of the observed recruitment problems in the PELICAN study, pain centers involved in the study as well as nonrecruiting pain centers were included. Furthermore, it aimed to highlight the positive and negative lessons learned from the conducted study and the number of obstacles the team had to overcome.

**Methods:**

A qualitative study, using a mixed methods approach, was performed. Multiple pain centers in Belgium completed an online survey, after which a structured interview was conducted to elaborate the responses in more detail. The broad topics of these meetings were feedback about the study, reviewing survey answers, and actions undertaken to enhance recruitment.

**Results:**

Different factors contributed to the low recruitment rate in the PELICAN study, such as limited and late referral from the general practitioners to the Belgian pain centers, insufficient internal referrals from nonpain specialists, lack of specific expertise on LNP in some centers, scarcity of staff, limited reimbursement to administer complex analgesic schemes, overestimation of the patient population, and the reluctance of patients to participate in pain research. Additionally, shortcomings in the implemented study design and the need for more logistical investments were identified.

**Conclusion:**

The findings of the qualitative study demonstrate the need for further, more varied LNP research in Belgium, not limited to pharmacological studies. It also sheds important light on the recruitment obstacles that may be faced during these studies. Future studies could support this research by offering better proposals for feasibility and recruitment, for instance, by designing and conducting a compelling pilot study or applying social media during the recruitment phase. *Clinical Trials*. This trial is registered with NCT03348735. EUDRACT number 2018-003617-17.

## 1. Introduction to the PrEgabalin Lidocaine Capsaicin Neuropathic Pain (PELICAN) Pragmatic Trial

Moderate to severe chronic pain occurs in 19% of European adults, significantly affecting the quality of their social and professional lives [1]. Epidemiological studies in Europe have indicated that 7%-8% of adults suffer from chronic pain with solid neuropathic characteristics when validated screening tools are applied. Based on the most recent population figures (January 1, 2022), this means that there are 926,720 potential Belgian patients with painful neuropathies. Peripheral neuropathies are in fact among the most common neurological diseases. Worldwide, a yearly incidence of 77/100,000 inhabitants is observed with a prevalence of 1%–12% in all age groups, but the incidence increases up to 30% in older adults [2–5]. When peripheral neuropathic pain affects a specific, clearly demarcated area of the body, it can be described as localized neuropathic pain (LNP). Examples include postherpetic neuralgia (PHN), painful diabetic polyneuropathy (DPN), as well as postsurgical pain. LNP accounts for up to 60% of all neuropathic pain syndromes [6]. It is a neuropathic pain condition of peripheral origin and is characterized by circumscribed areas of maximum pain coupled with abnormal skin sensitivity or spontaneous symptoms such as burning pain. To date, there are no specific guidelines for LNP management, so guidelines for general neuropathic pain have been used. Identification of patients with LNP facilitates an evidence-based therapeutic approach. Hence, specific studies on LNP, such as PrEgabalin Lidocaine Capsaicin Neuropathic pain (PELICAN), are of high clinical relevance.

**Figure 1:**
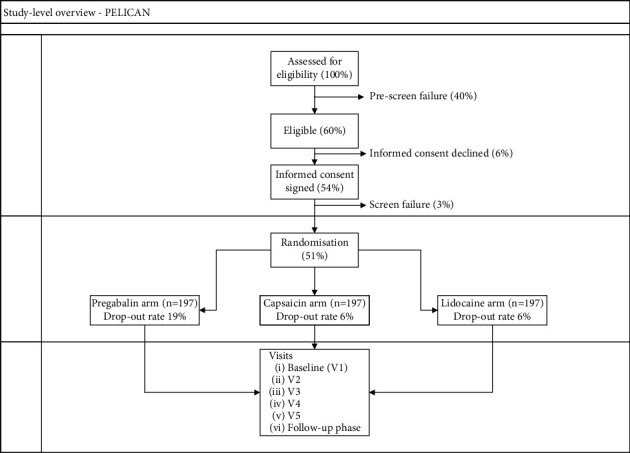
Overview of the study design of the PELICAN pragmatic trial as originally conceived.

The PELICAN study was a randomized, multicenter, comparative pragmatic trial that aimed to examine topical versus systemic treatment in adult patients with LNP syndromes. Systemic treatment options are often linked to side effects, whereas the topical route offers significant advantages. Notably, only a small fraction of the dose administered topically reaches the systemic circulation, thereby reducing the risk of systemic adverse effects, drug-drug interactions, and overdose. The Belgian Healthcare Knowledge Center (KCE) funded the PELICAN study under their KCE Trials program (see for more details on the program https://kce.fgov.be/en/kce-trials/funded-trials/pelican-localized-neuropathic-pain-a-study-to-compare-topical-treatment-versus-systemic-treatment). The PELICAN study aimed to compare two topical analgesic medications (lidocaine and capsaicin patches) with one of the commonly available systemic treatment options, i.e., the anticonvulsant drug pregabalin, for neuropathic pain conditions. The study was designed to compare topical and systemic medications according to the observed improvement in quality of life, pain relief, and occurrence of adverse effects. The study overview of the pragmatic PELICAN trial, as initially conceived including the initial recruitment, is detailed in Figure 1. Based on available scientific evidence, it was postulated that the two topical therapeutic options would be beneficial compared with the systemic treatment option from a side-effect point of view.

Recruitment for the PELICAN trial commenced in December 2018. Unfortunately, the recruitment of patients was extremely slow, and despite several actions to address this, the enrollment rate remained insufficient. Finally, and in mutual agreement, the project funder and sponsor decided to stop the trial on March 30, 2020, because of these inclusion difficulties and the COVID-19 pandemic. By the time of the study's premature closure, 32 patients had signed the informed consent form and had been randomized into 10 of the participating centers. All data have been registered in a synopsis report which can be accessed at https://kce.fgov.be/en/kce-trials/funded-trials/pelican-localized-neuropathic-pain-a-study-to-compare-topical-treatment-versus-systemic-treatment. The primary outcome analysis was still performed on the available data, but there was no significant effect of any of the treatment options on EQ-5D-5L (the 5-level version of the EuroQol multiattribute instrument for measuring health-related quality of life) at week 6. This absence of significance in the primary endpoint was expected due to the small number of patients. The lidocaine plaster group showed improved quality of life and pain relief during the initial phase of the trial, whereas the capsaicin-treated patients exhibited the improved quality of life and pain relief later in the trial. Although a limited number of patients were recruited, the demographic data corresponded with those of previous studies on peripheral neuropathies. This can be regarded as a confirmation of the study protocol validity and applied inclusion process.

One of the most challenging findings was the high discontinuation rate observed in both topical treatment options by week 26 (100% for lidocaine and 77.8% for capsaicin). This finding was unexpected, and unfortunately, the premature discontinuation of the trial prevented us from further identifying possible explanations for this finding. Regarding the observed safety profile, systemic treatment with pregabalin appeared to induce more adverse events than lidocaine treatment. Interestingly, the topical application of capsaicin patches seemed to also induce a substantial number of systemic adverse events.

Despite the observation of some interesting trends, in the end, there were insufficient data to draw any conclusions from the study, creating the need for a follow-up qualitative study to investigate the reasons for the unanticipated failure of this well-prepared trial. The funder and study team's main aspiration was to determine the reasons behind the recruitment failure through a scientifically based methodology. When a clinical trial is prematurely discontinued, it is essential that stakeholders take the right steps to ensure that lessons are learned, and trust in clinical research is maintained. However, this is often overlooked, leading to a lack of evidence on how recruitment failure occurs and its prevention. A recent study that investigated this delicate matter concluded that the decision to publish findings from a discontinued clinical trial should not rest solely on individual investigators but rather should be part of a systemic approach [7].

## 2. Methodology of the Qualitative Research Trial (Mixed Methods Approach)

A qualitative research project was conducted after the comparative trial was terminated. An explanatory sequential research design was hereby applied, in which quantitative data collection and analyses occurred first, followed by qualitative data collection and analyses [8–10]. This specific research design was chosen because the qualitative data are believed to explain and contextualize the quantitative findings. The quantitative data collection was obtained through a SurveyMonkey-based questionnaire, while the qualitative data collection was obtained by in-depth one-to-one interviews (video calls) with the principal investigators (PI) of the participating centers. Permission for the qualitative study was obtained from the Ethical Committee of the Antwerp University Hospital through an amendment to the permission of the PELICAN study.

An easy-to-fill online questionnaire was created and consisted of 25 items (for an example see: https://nl.surveymonkey.com/r/6NDCY7Q). The content of the questionnaire was beforehand validated for completeness by three members of the study team. After that, an expert panel of four independent judges (not previously linked to the conduct of the PELICAN trial) conducted content validity and reliability testing to assess the validity of the survey. To strengthen the overall validity of the survey, the experts paid special attention to the inclusion of concurrent (i.e., the correlation between information collected within the survey with information obtained from prescreening visits to the pain centers) and predictive validity measures (i.e., assessing the situation before the initiation of the PELICAN trial versus the situation after its termination) [11, 12]. Finally, its content and structure were discussed by all members of the scientific supervisory committee. Only after this final approval, the questionnaire was disseminated to the respondents. The survey was sent (digitally, via e-mail from SurveyMonkey) to all pain centers participating in the PELICAN study (*n* = 15). Answering all of the questions was mandatory to complete the survey. Instructions to correctly fill out the form were provided. Reminders were sent out twice (if no response was obtained earlier) to urge the centers to complete the survey. Within 8 weeks of the initial sending of the survey, completed questionnaires were obtained from all participating centers but one. After the completed questionnaire was received and responses recorded, an appointment was scheduled for an interview to discuss the responses and expand on these in more detail with the center's PI.

The digital survey (see Table 1) consisted of the following sections: (1) demographic data on the responding pain center, (2) knowledge and experience concerning the concept of LNP, (3) experiences related to the PELICAN study, and (4) post-PELICAN changes implemented in individual pain centers regarding the management of LNP based on their experiences during the PELICAN study. The interviews with the PIs from the participating centers were semistructured, so that experiences and attitudes could be explored [13]. Each interview was prepared individually, based on the center's respective responses to the digital survey. However, the interview also contained some questions developed in advance and presented to each participating center to standardize the interviews across different centers. A synchronous interview type was used because of the epidemiological circumstances owing to the COVID-19 pandemic; therefore, the interviews were conducted via videoconference (Teams or Zoom). All interviews were performed by the same investigator, with no previous relationship between the investigator and the interviewees, so no bias could occur. Interviews were not recorded, but the content of the interviews was saved in a predetermined template.

A slimmed-down version of the survey was also sent to accredited multidisciplinary pain centers in Belgium (a total of 35 RIZIV/INAMI accredited multidisciplinary pain centers), that had not previously participated in the PELICAN study. Several questions specific to the PELICAN study were hereby omitted, but the more general questions on LNP and its treatment were retained (see Table 1 for a detailed overview). It is highly relevant that pain centers previously not participating in the PELICAN study were also given the opportunity to complete the survey, so that a broad view of the nationwide situation could be obtained. Moreover, these centers often provided additional information on some practical drawbacks of specialized diagnostic and treatment protocols for LNP conditions (i.e., lack of personnel and lack of financing for the treatment with capsaicin). Responses from 11 additional pain centers were recorded. The additional centers were situated in the provinces of Antwerp (3), East Flanders (2), West Flanders (1), Limburg (1), and the French-speaking provinces Namur (1) and Liège (1) and the Brussels region (2). The responding pain centers typically apply a diverse range of noninvasive and invasive treatments.

Based on the feedback gathered from the participating pain centers during both the PELICAN trial and qualitative study, some crucial reasons for recruitment failure became apparent. These had not been identified while drafting the study protocol or during the feasibility study performed to assess this study's viability. These factors can be subdivided into study-related and more general causes related to the organization of pain management at the national level in Belgium. It is imperative that these findings be communicated and further elaborated on in a peer-reviewed publication considering their impact on public health. Furthermore, these findings should be accessible to the broader public and known to other research groups, so that they can be identified and addressed during the conception of future trial protocols.

## 3. Findings

The qualitative study revealed several different reasons for the low recruitment rate. One of the reasons was the somewhat limited clinical trial experience in approximately half of the participating pain centers. Responses varied that centers performed one to eight clinical trials every 5 years. Furthermore, a significant proportion of these studies were related to somatic or mixed (neuropathic-somatic) pain syndromes rather than neuropathic pain alone. Neuropathic pain studies have almost exclusively been limited to either PHN or DPN. This shows that research experience regarding LNP conditions is rather limited in Belgium. Nevertheless, 33% of the responding centers claimed to have previously conducted one or more LNP-specific studies. Most research experiences were in the context of pharmacological studies, and these studies were dated to at least 3 years ago. In addition, two-thirds of the responding pain centers claimed to have experience in conducting pragmatic trials, but during the interviews, it became clear that while pragmatic studies are well known, some studies are mistakenly defined as pragmatic by investigators. Therefore, actual experience with pragmatic studies is lower than initially assumed.

Another possible reason for the low recruitment lies in the nonavailability of dedicated study staff. Sixteen of the responding pain centers declared that they had specific staff available to conduct clinical trials. However, it was discovered that this availability was mainly on a part-time basis. The other centers, however, did not have any dedicated study staff. This is understandable considering the small number of algology (pain treatment) clinical studies recently conducted, but this significantly negatively impacts the recruitment of patients in clinical studies.

**Figure 2:**
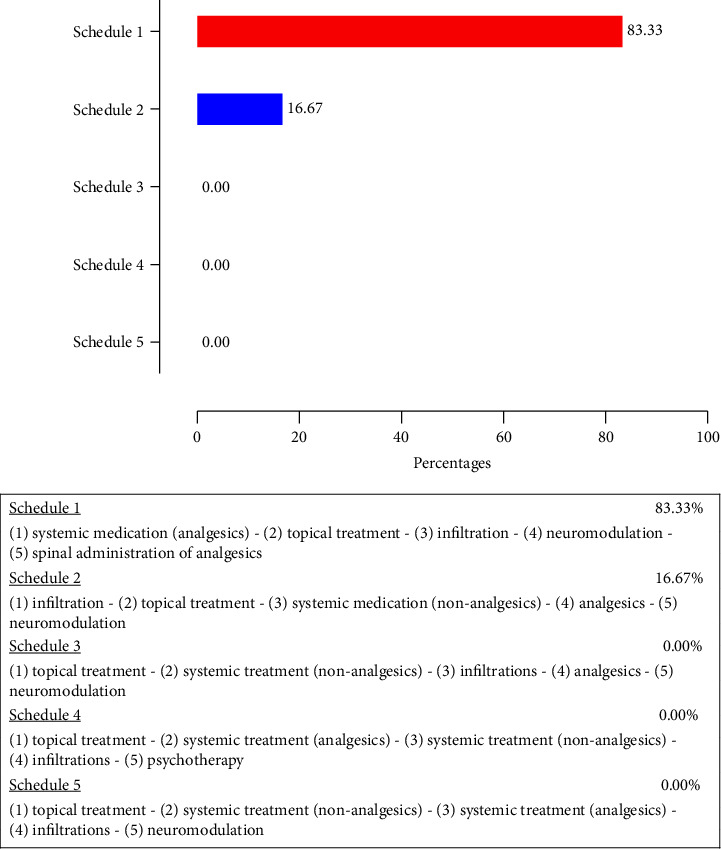
Different flowcharts that were evaluated in the survey as possible first-choice treatment approaches for localized neuropathic pain conditions within Belgian multidisciplinary pain centers. Topical treatment options are never considered as a first-line treatment in these conditions.

Furthermore, it became clear that many centers have no specific treatment protocols available for LNP. When different options were presented during the digital survey, 83.33% of the responding pain centers identified the following flowchart as their first-choice treatment approach for LNP conditions: (1) systemic medication (analgesics), (2) topical treatment, (3) infiltration, (4) neuromodulation, and (5) spinal administration of analgesics. The different flow charts that were proposed for review are detailed in Figure 2. This finding clearly indicates that LNP syndromes are currently treated following treatment guidelines for general neuropathic pain.

Recruitment appeared slow from the outset and relied primarily on internal referrals. This raises the question as to why there are so few external referrals to pain centers. Interviews revealed that general practitioners (GPs) tend to treat LNP complaints themselves, mostly applying systemic treatment options (such as pregabalin). Thus, many patients had already completed pregabalin treatment before referral to the pain center, impacting their inclusion into the study protocol. In addition, patients tend to remain in primary care or related specialties for a longer period, so late referral also contributed to the low recruitment rate, with many patients falling outside the set time frames for the duration of pain symptoms. Such a high level of late referral had not been anticipated by the PELICAN study team or local investigators during the feasibility studies.

Notably, 50% of the patients who were prescreened refused to participate in the study—higher than initially anticipated—due to patients' apprehension about financial implications. This is because, initially, only the systemic option (pregabalin) was reimbursed. During the recruitment period, the high concentration capsaicin patch, was reimbursed. Patients could now choose a fully reimbursed topical treatment option, causing a significant number of patients (general estimate across the different pain centers: at least 30 patients) to become reluctant to participate and rather “safely” opt for the reimbursed capsaicin treatment.

Participating centers also mentioned insufficient reimbursement for nursing activities related to the application of the concentrated capsaicin patches. Treatment with topical capsaicin requires a significant time investment from pain nurses, without any compensation or lump sum reimbursement. Although a substantial additional compensation was provided by the study protocol for the execution of capsaicin treatment by the funder, this was still perceived as too little by some participating centers. Undoubtedly, this had an additional negative impact on recruitment. Interestingly enough, a similar situation has arisen in routine clinical practice. After the capsaicin patches were reimbursed by the social security system in Belgium, uptake in clinical practice remained limited due to nonreimbursement for nursing activities. The main reasons for recruitment failure in the PELICAN study are described in more detail in Table 2.

A specialized monitoring system was developed so that patients and investigators could submit all data and assessments related to the PELICAN study digitally. The PELICAN@home platform—a primary source—was hosted on servers within the central server facility at the Antwerp University Hospital. During the trial, feedback was received from participating pain centers that some patients faced difficulties in completing the digital assessments. Therefore, the study team ensured that all assessments were also available on paper (even in different languages). Nevertheless, during the digital survey, half of the PIs described the digital solution as too complex. The other half rated the digital platform as rather difficult (16.7%) or acceptable (33.3%). No PI rated the PELICAN@home as user-friendly. From the patients' perspective, a slightly different picture emerges as no one considered the platform to be too complex. Therefore, the digital platform was considered quite complex by and for the patients, but within acceptable standards. Although it was not clear from the survey that using the digital platform caused anxiety in patients, pilot testing of the user-friendliness of implemented digital solutions must be considered in future trials.

Four primary reasons for the slow recruitment rate were identified in the qualitative study as follows:

1. Topical treatment of LNP is underutilized in clinical practice. This should be viewed against the background of less attention paid to the application of less invasive pharmacological versus the more invasive treatment options within the pain center.
2. Referral of patients suffering from LNP (and pain conditions in general) from primary care to multidisciplinary pain centers is slow and often occurs only after other specialties have been repeatedly consulted. Acute or subacute pain syndromes are rarely observed in tertiary pain centers. Therefore, future studies in this field should be firmly anchored with primary care to enroll these patients on time.
3. Some study protocol-related criteria negatively impacted recruitment (e.g., a ban on the previous use of gabapentinoids).
4. Finally, some pain patients, as well as caregivers, proved to be less digitally savvy than expected, so a paper backup system had to be provided.

## 4. Discussion

A majority of patients with neuropathic pain suffer from LNP. Based on the International Association for the Study of Pain (IASP), LNP is described as a type of peripheral neuropathic pain characterized by consistent and circumscribed area(s) of maximum pain associated with abnormal sensitivity of the skin and/or spontaneous symptoms characteristic of neuropathic pain [14, 15]. By successfully identifying patients with LNP, this definition facilitates an evidence-based approach to neuropathic pain management. However, this is not always easy to perform in routine clinical practice. To support clinicians in this challenging task, an identification tool for LNP was developed and validated [16]. The tool was shown to be accurate in distinguishing between LNP and nonlocalized neuropathic pain conditions with a sensitivity of 53.2% and a specificity of 88.2%.

Regarding the initiative on methods, measurement, and pain assessment in clinical studies, several outcome measures, corresponding to six domains, have been recommended for assessment in pain trials: (1) pain, (2) physical functioning, (3) emotional functioning, (4) participant ratings of improvement and satisfaction with treatment, (5) symptoms and adverse events, and (6) participant disposition. However, since many treatments often only provide meaningful pain reductions in 40%–60% of patients, other outcomes such as physical, social, and occupational function are sometimes considered to have comparable importance [17]. Therefore, in the PELICAN study, the impact on quality of life, aside from physical function such as pain reduction, lifestyle changes, and adverse events, were chosen for evaluation to compare one systemic and two topical medications and their effects.

However, the results of the preliminary literature review on LNP revealed a lack of conclusive research concerning topical therapeutic options. A limited number of studies discussed and compared the efficacy of each of the topical treatments with that of a placebo. However, a 2014 systematic review of topical lidocaine for neuropathic pain (involving 280 patients with PHN) showed very low-quality evidence of the efficacy of topical lidocaine owing to small sample size, incomplete outcome assessments, and modest outcome measures of efficacy. Other clinical trials have found that 5% lidocaine patches are effective and well-tolerated for treating PHN with minimal toxicity risk or drug-drug interactions [18, 19]. A recent study investigating topical treatment with lidocaine-medicated plasters for LNP conditions in routine clinical practice showed that the lidocaine treatment provided significantly greater improvement in pain-related impairments of daily living and quality of life than oral antineuropathic medications [20].

The capsaicin dermal patch delivers a high concentration (8% w/w) of synthetic capsaicin, a highly selective agonist of transient receptor potential vanilloid-1 (TRPV-1), directly to the site of pain. The 8% capsaicin dermal patch is indicated in the European Union (EU) for the treatment of peripheral neuropathic pain in adults, either alone or in combination with other medicinal products [21]. In the US, the patch was recently also approved by the FDA for the treatment of DPN [22]. In patients with DPN, a single 30-min application of the 8% capsaicin dermal patch provided 12 weeks of pain relief and improved sleep quality compared with placebo [23]. Repeat treatment with the 8% capsaicin dermal patch in diabetic patients over 52 weeks provided sustained pain relief, without additional side effects compared with standard care alone [24]. The 8% capsaicin dermal patch was noninferior to oral pregabalin in relieving pain in patients with nondiabetic PNP, with a faster onset of action and greater treatment satisfaction [25]. A single 60-min application of the 8% capsaicin dermal patch provided rapid and sustained pain relief in patients with PHN [26]. Results in patients with HIV-associated neuropathy were somewhat contradictory, with a significant improvement in pain intensity observed in one trial, but not in the other [27, 28]. An expert opinion stated that in cases of LNP, evidence supports a pragmatic approach of using a local treatment before considering a systemic treatment [29]. Furthermore, the capsaicin patch was shown to be cost-effective compared with dose-optimized pregabalin in patients with PNP with refractory systemic treatments [30].

Considering the current lack of solid scientific evidence, the findings of the PELICAN study would have provided valuable and unique information regarding the comparison between systemic and topical medications. Despite the extensive preparation and review of the final study protocol, the PELICAN study had to be discontinued due to the low recruitment rate. Some possible primary causes were revealed during the qualitative study, but it is also necessary to critically assess the reasons for the termination of the PELICAN study considering the observed findings in other unsuccessful studies.

Failure to enroll sufficient participants in a trial has been a long-standing problem [31]. An overview of 114 UK trials indicated that only 31% met the enrollment goals. Another study reported that one-third of publicly funded trials required time extensions because they failed to meet the initial recruitment goals [32]. A study from Switzerland found that recruitment problems caused premature discontinuation in one of four randomized clinical trials (RCTs) supported by the Swiss National Science Foundation. Furthermore, 40% of RCTs (70% of discontinued RCTs) were never published in a peer-reviewed journal. RCTs are the cornerstone in the evaluation of preventive and therapeutic healthcare interventions. However, empirical evidence suggests that 20%–25% of initiated RCTs are prematurely discontinued, with poor participant recruitment as the main underlying reason [33]. A retrospective cohort study investigating the premature discontinuation of pediatric RCTs even came up with a discontinuation rate of 40% [34]. The recent COVID-19 pandemic also had some drastic impact on medical practice and on the conduct of clinical research. A recent survey found that some 16.4% of anesthesiology-related clinical trials were discontinued within the designated data range due to COVID-19-related issues [35].

In the PELICAN trial, recruitment appeared slow from the outset and relied primarily on internal referrals (within the participating hospitals). Although educational material and flyers were kept at the disposal of the pain centers, it remained difficult to substantially increase the volume of internal referrals in all participating centers. In addition, the study team quickly observed that almost no patients were referred to the participating pain centers by primary care practitioners. It is commonly known that most chronic pain patients are treated by GPs. The previously mentioned large-scale European pain survey by Breivik et al. indicated that most pain patients (70%) are treated by GPs and 27% by an orthopedic specialist. Only 2% of chronic pain patients were treated by a pain management specialist. Most respondents (69%) had been treated by the same physician for 1–15 years. When explicitly asked whether they had ever been to a pain management specialist, 23% of patients (mean) responded affirmatively. This percentage, however, differed significantly throughout Europe, ranging from 8% in Norway to 40%–43% in France, Israel, and Italy. It is obvious that every pain-related study must involve GPs in the inclusion of patients and to enable timely referral of sufficient numbers of pain patients. Failure to actively involve GPs in the design and execution of the study protocol will significantly compromise a successful recruitment strategy.

Efforts were made to address the recruitment issues: a substantial number of GPs in all geographical parts of Belgium received a letter explaining the trial, patient flyers were provided for the waiting room, the trial was made public via a website and social media, and the study team presented the PELICAN study on different occasions to both pain and nonpain colleagues. Unfortunately, none of these efforts had the desired results since no impact could be observed on the recruitment rate.

After discussing this lack of recruitment with the participating clinics' representatives, the PELICAN study team revised the target sample number. The feasibility report elaborated on each center's capability to recruit patients and the period needed to accomplish this. Later, the report was discussed again with the investigators, who agreed on the selected target, yet the slow recruitment rate still did not improve. Thus, there was a considerable gap between the actual and intended patient numbers. Therefore, overestimation could have played a role in this study. It is crucial to properly understand how large the corresponding patient population is, how many people are needed in this population, and how many will participate. This is where many investigators overestimate the number of patients to be ultimately included in their trial [36].

The inclusion and exclusion criteria are important for the reliability of medical trials. Very restrictive criteria cause willing patients to be excluded for not fulfilling the trial's essential criteria. Likewise, some of the inclusion and exclusion criteria applied in the PELICAN study may have affected the number of patients included in the study. It became evident that pain duration was often prolonged in patients who were prescreened for inclusion in the PELICAN study. Unfortunately, this is in accordance with general epidemiological findings. Breivik et al. showed that only 12% of respondents have suffered from pain for <2 years, almost 60% between 2 and 15 years, and many (21%) had lived with pain for ≥20 years, with an overall median pain duration of 7.0 years [37]. Considering this information with respect to the PELICAN study's inclusion criteria—the presence of pain for 3–24 months—it appears this inclusion criterion was overly restrictive.

Another protocol-related criterion that negatively impacted recruitment was related to the exclusion of previous treatment with gabapentinoids. This proved to be especially critical as the study started from pain management clinics and not from general practices. Considering the common treatment trajectory of patients with pain, many had already been introduced to analgesics and antineuropathic drugs and were therefore no longer eligible for enrollment. Primary care physicians are accustomed to using anticonvulsant drugs in patients with suspected neuropathic pain conditions. Due to the restrictive nature of the study protocol, even a short-term trial therapy with an anticonvulsant immediately resulted in exclusion from the PELICAN study, even if no topical treatment had previously been tested. In the future, one should consider implementing changes to the inclusion criteria for analgesic trials so that short-term negative trial treatments can be discarded based on several predetermined criteria such as duration of trial treatment, the maximum dose administered, and occurrence of side effects. Such an approach would increase the number of patients eligible for inclusion in the study protocol.

We should ask ourselves whether recruitment failure could have been prevented. Briel et al. stated that the most common reasons for recruitment failure seem preventable in a pilot study that applied the planned informed consent procedure [38]. This was not considered in the PELICAN trial since there have been studies using lidocaine plasters and capsaicin patches in Belgium. However, and this is perhaps a crucial element, Belgian pain centers participated alongside international pain centers in those studies. Therefore, the burden of recruitment was spread across several countries. The need for performing a pilot study for the feasibility of recruitment in pain-related trials has been proposed in other specific study settings, such as general practices [39, 40].

Another reason for recruitment failure in the PELICAN study could be linked to the irregular medical follow-up by chronic pain patients. This was mentioned in several interviews during the qualitative study and is confirmed by the findings of previous studies. Breivik et al. in their European survey on chronic pain delved more closely into the number of patients' visits to doctors. The interviewers asked participants how many times in the last 6 months had they visited their primary treating physician for their illness or medical condition that caused pain. Reportedly, 16% had not visited their physician at all, 14% had visited only once, 60% had visited two to nine times, and 11% had visited at least 10 times in the last 6 months. Of the respondents, 35% had consulted one single physician, while 54% had consulted two to six different physicians during the same time frame [37]. This means that, to optimize inclusion in pain trials, investigators should also directly target patients to obtain better recruitment. Cooperation with patient organizations is therefore mandatory since they can facilitate in delivering information on ongoing studies directly to patients.

Several concerns were raised regarding several digital aspects of the study. Digital health has become more important, especially since the COVID-19 pandemic, but it should be implemented with a lot of considerations for patients as well as for the supporting study staff. Half of the responders from the participating pain centers believed that using the digital platform for the follow-up of the therapeutic response and adverse events caused anxiety in some patients or even dissuaded them from (further) participation. This feedback is of major concern for the study team and is somewhat unexpected since the application of a similar digital platform in previous pain-related studies was well received by patients [41, 42]. Additional pilot testing in the targeted patient population could perhaps have solved many problems before the study initiation. The demographics of the study population also possibly induced a higher degree of nontech savviness, precluding these patients from fully absorbing the digital features of the follow-up. Notably, there was a high unemployment rate in the PELICAN study group (44.4%–70% in different groups). Additionally, the digital medication quantification scale (MQS, version 3) proved to be particularly challenging for both patients and physicians. Although this medication evaluation tool has already been used in other pain-related databases, its correct implementation has proven challenging in the specific context of the PELICAN study [43–45]. This is a further indication of the fact that the specific patient population, which was included in the PELICAN study, had critical issues in using the applied digital monitoring system. The use of pilot phases, user feedback groups, and gamification techniques to further optimize the user experience should become part of future pain trials [46, 47].

Patients may experience out-of-pocket costs when participating in a clinical study, including transportation costs and lost work as well as medical costs for additional testing or interventions. Insurance or social security may not cover medical care beyond what is deemed evidence based. Even when medical care is covered, deductibles are often relatively high, and a patient may not be able to afford participation. Notably, patients' fear of having to pay for the costs themselves after the study ends significantly hampered their willingness to participate in clinical trials [48]. Pain patients are particularly concerned about this financial aspect because of their often-precarious socioeconomic situation and the chronicity of their pain condition. This aspect was clearly mentioned in the qualitative study. Although study-related costs were entirely covered by the funder, many patients remained reluctant to participate in the PELICAN trial since two of the three treatment options were not reimbursed in Belgium at the start of the recruitment period. Patients feared that they would have to pay for the continuation of the treatment at the end of the study period if reimbursement remained unavailable at that time. When, during the study, reimbursement became available for a second study medication (the highly concentrated capsaicin patch), patients preferred to try this treatment under regular reimbursement rather than going through a randomization process and being at risk of getting allocated to a nonreimbursed treatment option (such as the lidocaine-medicated plaster). This negatively impacted the recruitment rate. Within trial programs, funders and sponsors should consider including post-trial provisions for continued financing of nonreimbursed, but successful, therapies for a defined duration after the end of the regular study duration. This is also in line with the Declaration of Helsinki §34 (https://www.wma.net/policies-post/wma-declaration-of-helsinki-ethical-principles-for-medical-research-involving-human-subjects/) regarding post-trial provisions. This would allow for successful treatments to obtain regular reimbursement based on the study findings, while economically fragile patients would be assured that they would never have to pay the full cost of treatment.

One additional comment should be made regarding the capsaicin treatment that could have negatively impacted patients' willingness to participate in the trial. Patients are unfamiliar with capsaicin as a therapeutic agent [49]. The 1 h application of capsaicin patches induces short-lasting additional pain complaints (burning sensation), which can, in turn, lead to ingrained fear in patients [21]. Even though study personnel was instructed to clearly explain to patients that these additional pain complaints were transient and administration of additional analgesic drugs could be requested, patients' fear of such procedure-related increase in pain was perhaps overlooked and/or underestimated by the trial team. According to previous findings, patients often experience additional pain caused by further treatment and are in fear of such pain. Patients appeared more motivated if they knew from prior clinical encounters what to expect. This indicates that education is essential for patients, but sometimes underestimated by physicians and supporting staff during the entire process, hinting at some mistrust or disappointment from patients toward medical interventions [50].

Investigators should also carefully consider which study method is the most appropriate to obtain the necessary information. Qualitative surveys, like the one we performed, are an often-used possibility, but one should also consider the performance of Delphi studies. Surveys have been performed in several countries to investigate some major pain-related issues [51–54]. In Italy, a nationwide survey was conducted to investigate the strengths and weaknesses of cancer pain management in the country [55]. Another survey investigated the state of pain in the elderly with dementia throughout Europe, hereby considering assessment, treatment guidelines as well as global policies for this specific patient population [56]. Another method to perform large-scale surveys is the application of Delphi investigations. Delphi studies can be used to identify the optimal diagnostic criteria, such as for rotator cuff-related shoulder pain [57]. However, the Delphi methodology can also be applied to investigate therapeutic interventions. A recent Delphi study investigated the management of cancer-related neuropathic pain in Spain, indicating some disagreement on the multidisciplinary approach and referral criteria of such patients [58]. A multiple round Delphi protocol was also applied to explore new diagnostic criteria for opioid use disorders [59]. The availability of e-Delphi surveys allows for the inclusion of participants from many different countries into a single project, even when applying different rounds [60].

Finally, there is also some critical discussion outlining the role and possible benefits of social media in augmenting and supporting the original methods of recruitment. Reuter and Lee investigated this matter and concluded that, mostly, social media has complemented rather than replaced traditional recruitment efforts [61]. Nevertheless, the use of social media has the potential to accelerate the timeline toward achieving accrual targets. In frail patients with (chronic) pain, this is undoubtedly an aspect that must be considered in future clinical projects to enhance recruitment. However, the authors rightly noted that managing ethical and regulatory aspects will be necessary to take full advantage of this novel opportunity [62]. Darko et al. published in their recent review a first guideline to be used by ethical boards and research support services within academic institutions to provide guidance for researchers to effectively use this recruitment method [63].

All the abovementioned findings are essential to be taken into consideration in future pain studies. Given the extremely low number of drugs currently approved for pain treatment, it is crucial to address all barriers to recruitment to allow new therapies to be successfully validated in completed clinical trials. For instance, the same remarks have been formulated for studies related to head and neck cancer [64].

On the positive side, the topical administration of capsaicin has become more widely known within pain centers because of their participation in the PELICAN study. Moreover, in half of the participating pain centers, participation in the PELICAN study was used to facilitate the clinical diagnosis of LNP.

## 5. Conclusion

The participating pain centers considered low enrollment in the PELICAN study, mainly due to insufficient (timely) referral from primary healthcare providers and other specialties, to be the main issue leading to the study's premature cessation. Additionally, poor recruitment was also attributed to the too strict inclusion and exclusion criteria—limiting the inclusion of many patients with neuropathic pain. Third, the reimbursement of high-concentrated capsaicin patches, which was granted after the initiation of the PELICAN study, undoubtedly further reduced the inclusion rate. Patients opted for the reimbursement treatment option rather than going through the burden of the study protocol and uncertainty about the follow-up treatment after the study completion.

Based on the findings of this qualitative study, 10 points should be considered important for health policy at the national level as well as for future studies within the specialism of pain treatment as follows:

1. Topical treatment of localized neuropathies often remains (somewhat) unknown (thus, not considered in the treatment regimen).
2. Topical treatment modalities are inadequately used in clinical practice because of reimbursement modalities. This has significantly hampered the introduction of such treatment modalities in routine clinical practice.
3. Faster detection and referral of patients with LNP from primary healthcare providers or other medical specialties to specialized pain centers.
4. Direct inclusion of patients from primary healthcare providers with the active participation of GPs in the development of protocols and recruitment.
5. Training primary healthcare providers and physician specialists to make correct diagnoses (recognition of pain syndromes such as LNP).
6. A better description of the distinction between subacute pain syndromes and chronic pain syndromes and possibly including the two separate cohorts in a future study protocol.
7. Better support should be provided for pain centers with a less well-developed study team, with consideration given to possible financial reimbursement for nursing actions as part of the study.
8. Fundamentally, the problem of an unbalanced focus on invasive treatment techniques versus pharmacological or noninvasive treatment options arises. This is undeniably linked to the underlying funding systems—or lack thereof.
9. Further, simplified and rationalized digital platforms and tracking systems and accommodation of the still significant digitally illiterate group.
10. Need for innovation in pain treatment. There is concern regarding the lack of development of new therapeutic agents or approaches to acute and chronic pain. Therefore, there is a lack of experience in performing applied research in pain centers.

The findings of the qualitative study demonstrate the need for further, more varied LNP research in Belgium, not limited to pharmacological studies. It also sheds important light on the recruitment obstacles that may be faced during these studies, which can be overcome in subsequent research by using or at least considering some of the information presented in this paper. Support programs by study sponsors might be helpful for investigators involved in discontinued trials and promote transparency and learning lessons for future studies. Furthermore, our qualitative study explores the various methodologies that may help recruit patients and participants ahead of trials. Future studies may aid this research by offering better proposals for feasibility and recruitment, for instance, by designing and conducting a compelling pilot study or applying social media during the recruitment phase.

## Figures and Tables

**Table 1:** Content of digital survey that was provided to the respondents. The questionnaire contained 3 major parts: (1) gathering general information on the pain centers that completed the survey, (2) focusing on specific information regarding the Pelican trial, and (3) collecting information on future considerations concerning the diagnosis and treatment of localized neuropathic pain syndromes.

Sections	General content (number of questions to be completed by respondents)	Specific items questioned	Completed by centers participating in the PELICAN trial	Completed by centers not participating in the PELICAN trial
Part 1	General information on the pain center (*n* = 8)	Geographical location pain center	Yes	Yes
		Experience in pain studies during the last 5 years (and number of studies)		
		Experience in neuropathic pain-related studies (and type of investigational intervention)		
		Previous participation in LNP-related studies		
		Experience in pragmatic studies		
		Availability of study personnel and infrastructure		

Part 2	Trial specific information (*n* = 13)	Main reasons for recruitment failure (multiple answers possible)	Yes	No
		Which inclusion criteria were most obstructive to inclusion?		
		Wrong comparator chosen		
		Impact of postulated limitation in duration of LNP		
		Type of referral patterns present (and different for LNP conditions)		
		Treatment protocols for LNP: which ones?		
		Treatment protocols in accordance with the proposed protocols in the study protocol		
		Ease of use digital platform (patients)		
		Ease of use digital platform (caregivers)		

Part 3	Follow-up considerations on LNP (*n* = 4)	Changes in clinical practice since 2020	Yes	Yes
		Changes in treatment options and analgesic protocols for LNP		
		New topical treatment options introduced for LNP		
		Changes in referral patterns		

**Table 2:** Main reasons for recruitment failure in the PELICAN study, as collected during the qualitative study that was performed after the closure of the pragmatic study. The mentioned reasons are displayed in descending order of importance, as indicated by the surveyed pain centers.

Categories of answers (reasons for recruitment failure)	(%)
Refusal by patient	50.00
No patients meeting inclusion criteria	50.00
Exclusion criteria too severe	50.00
Patients previously treated with one or more study medications	50.00
Reimbursement already available for study medications	33.33
No patients with LNP	33.33
Clinical practice too busy to include patients	16.67
Study protocol too complex	16.67
Insufficient possibilities to apply capsaicin in clinical practice	16.67

## Data Availability

The data from the pragmatic study are available through the website from the funder (KCE Clinical Trials). The link to the website and the research synopsis report is also mentioned in the manuscript: https://kce.fgov.be/en/kce-trials/funded-trials/pelican-localized-neuropathic-pain-a-study-to-compare-topical-treatment-versus-systemic-treatment.
